# Association of Prediabetes and Recurrent Stroke in Atrial Fibrillation Patients: A Population-Based Analysis of Hospitalizations and Outcomes

**DOI:** 10.3390/jcm13020573

**Published:** 2024-01-19

**Authors:** Rupak Desai, Advait Vasavada, Bhavin A. Patel, Maharshi Raval, Avilash Mondal, Kshitij Mahajan, Nishanth Katukuri, Yash Varma, Akhil Jain, Geetha Krishnamoorthy

**Affiliations:** 1 Independent Researcher, Atlanta, GA 30033, USA; drrupakdesai@gmail.com; 2 Department of Family Medicine, University of Nebraska Medicine, Omaha, NE 68198, USA; advait2163@gmail.com; 3 Department of Internal Medicine, Graduate Medical Education, Trinity Health Oakland Hospital, Pontiac, MI 48341, USA; bhavin.patel@trinity-health.org (B.A.P.); kshitij.mahajan@trinity-health.org (K.M.); geetha.krishnamoorthy@trinity-health.org (G.K.); 4 Department of Internal Medicine, Landmark Medical Center, Woonsocket, RI 02895, USA; 5 Department of Internal Medicine, Nazareth Hospital, Philadelphia, PA 19152, USA; avilashmandal98@gmail.com; 6 Department of Internal Medicine, Mayo Clinic, Rochester, MN 55905, USA; katukurinishanth553@gmail.com; 7 Division of Cardiovascular Medicine, Graduate Medical Education, Trinity Health Oakland Hospital, Wayne State University, Detroit, MI 48202, USA; yash.varma@trinity-health.org; 8 Department of Leukemia, The University of Texas MD Anderson Cancer Center, Houston, TX 77030, USA; akhiljaindr@gmail.com

**Keywords:** prediabetes, atrial fibrillation, stroke, recurrent stroke, mortality, cost

## Abstract

Prediabetes is a risk factor for ischemic stroke in atrial fibrillation (AF) patients, yet, its impact on recurrent stroke in AF patients remains understudied. Using the 2018 National Inpatient Sample, we investigated the link between Prediabetes and recurrent stroke in AF patients with prior stroke or transient ischemic attack (TIA). Among 18,905 non-diabetic AF patients, 480 (2.5%) had prediabetes. The prediabetic group, with a median age of 78, exhibited a two-fold higher risk of recurrent stroke compared to the non-prediabetic cohort (median age 82), as evidenced by both unadjusted (OR 2.14, 95% CI 1.72–2.66) and adjusted (adjusted for socio-demographics/comorbidities, OR 2.09, 95% CI 1.65–2.64, *p* < 0.001). The prediabetes cohort, comprising more male and Black patients, demonstrated associations with higher Medicaid enrollment, admissions from certain regions, and higher rates of hyperlipidemia, smoking, peripheral vascular disease, obesity, and chronic obstructive pulmonary disease (all *p* < 0.05). Despite higher rates of home health care and increased hospital costs in the prediabetes group, the adjusted odds of all-cause mortality were not statistically significant (OR 0.55, 95% CI 0.19–1.56, *p* = 0.260). The findings of this study suggest that clinicians should be vigilant in managing prediabetes in AF patients, and strategies to prevent recurrent stroke in this high-risk population should be considered.

## 1. Introduction

In recent years, the intersection of AF and metabolic health has emerged as a focal point in cardiovascular research. Notably, prediabetes, a condition where blood glucose levels are higher than normal but not high enough to be diagnosed as diabetes [1], has garnered attention for its potential role as an independent risk factor in non-diabetic AF patients. While previous studies have linked prediabetes to various cardiovascular events [2], the specific association between prediabetes and recurrent stroke in individuals with AF remains a subject of investigation. By scrutinizing a large national database, we seek to contribute valuable insights that extend beyond the established realms of AF management, ultimately guiding future strategies for risk assessment, intervention, and comprehensive patient care. 

Ischemic stroke is a significant cause of morbidity and mortality worldwide, and atrial fibrillation (AF) is a known risk factor for this condition [3]. AF is the most common cardiac arrhythmia, and it is estimated that approximately one-third of patients with AF will experience a stroke at some point in their lifetime. The pathophysiology of stroke in AF is attributed to the formation of blood clots in the heart chambers, which can then travel to the brain and cause ischemic injury. Despite the availability of effective therapies for stroke prevention, AF-related strokes continue to occur, and understanding the risk factors for stroke in AF is critical to guide therapeutic decision-making [4]. Prediabetes can affect arteries through multiple molecular mechanisms, including impaired insulin signaling, inflammation, oxidative stress, activation of the RAAS, and endothelial progenitor cell dysfunction [5]. Hence, the molecular mechanisms theoretically impact those with pre-existing cardiovascular disease. Recently, prediabetes has even been found to increase the risk of myocardial infarction. Hence, we aimed to study the impact of prediabetes on atrial fibrillation [2,6]. Specifically, we conducted this population-based study that will examine the role of prediabetes in particular and co-morbidities such as age, hypertension, diabetes, prior stroke or transient ischemic attack (TIA), heart failure, and coronary artery disease in AF-related stroke patients. The findings of this study will provide a comprehensive understanding of the risk factors for ischemic stroke in AF, which can help inform clinical management strategies for this high-risk population. Healthcare providers can use this information to improve patient care and outcomes through targeted screening, management of comorbidities, and optimization of healthcare resource utilization.

## 2. Materials and Methods

### 2.1. Data Sources

The study utilized the National Inpatient Sample (NIS) database for the year 2018, which is a part of the Healthcare Cost and Utilization Project (HCUP) sponsored by the Agency for Healthcare Research and Quality [7]. NIS is the largest all-payer inpatient healthcare dataset in the United States, representing about 20% of United States hospitals from 48 states, comprising an average of 7 million unweighted discharges per year that approximate more than 35 million weighted nationwide discharges. The database provides one primary diagnosis and up to 24 secondary discharge diagnoses for each inpatient admission. As the NIS database contains deidentified data, Institutional review board approval was not necessary. Additional information about the database can be accessed from the HCUP website. 

### 2.2. Study Participants

**Figure 1:**
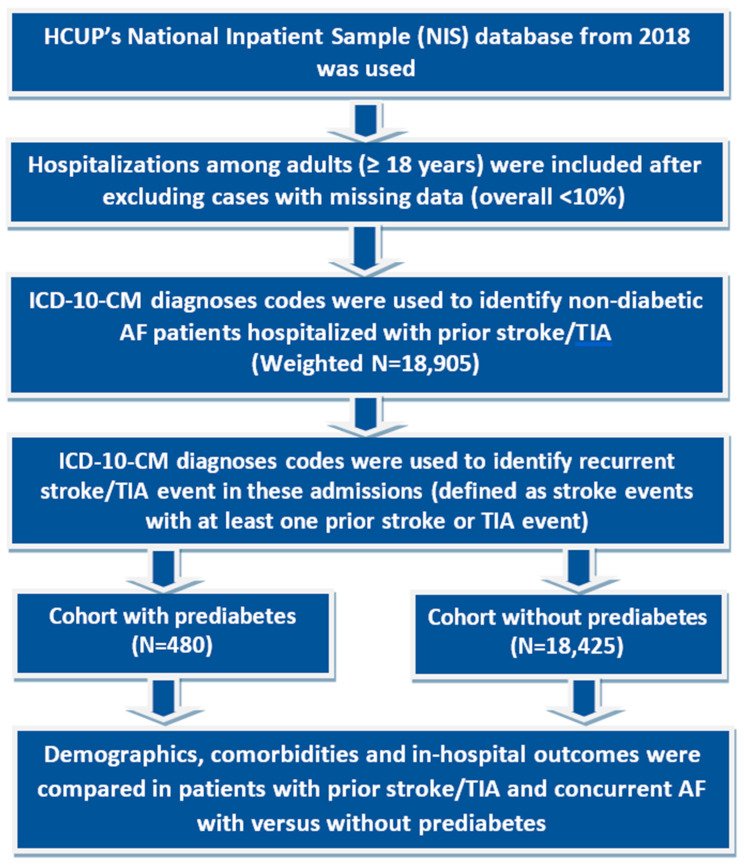
Flow diagram showing exclusion criteria and how the final study cohorts were reached. AF: Atrial fibrillation.

Patients with prediabetes (ICD code: R73. 03) and AF (ICD codes: I48.0, I48.1, I48.2, I48.3, I48.4, I48.9) with prior stroke/TIA were identified using relevant ICD-10 CM codes. Patients with AF were divided into two groups consisting of those with and without prediabetes, respectively, excluding the diabetic population. Patients with both prediabetes and AF were considered the exposure group and the other group was considered the control. Then the co-morbidities and outcomes are identified using Revised Clinical Classification Software codes. Cardiovascular and extracardiac comorbidities were determined by utilizing the Elixhauser comorbidity indices. Predefined criteria were found in the NIS database, which are based on ICD-10 CM codes (Figure 1). 

### 2.3. Study Outcomes

The primary objective of the study was to determine the relationship between prediabetes and recurrent stroke in non-diabetic atrial fibrillation (AF) patients with a history of stroke or transient ischemic attack (TIA). Recurrence of stroke was defined by current stroke admissions with at least one prior stroke/transient ischemic attack event using ICD-10 codes. The secondary objectives were length of hospital stays, hospital costs, and comorbidities related to AF hospitalizations.

### 2.4. Statistical Analyses

Descriptive statistics were used to describe the study population and initial characteristics were obtained. Categorical variables and continuous variables were reported as frequency and percentage, interquartile ranges, respectively. Pearson’s chi-square test and Mann–Whitney U tests were utilized for categorical and continuous variables (non-normal distribution), respectively, to compare baseline demographics, hospital characteristics, and other comorbidities between the two groups. The discharge weight provided in the database was used to generate national estimates. To evaluate the relationship between prediabetes (pDM) and recurrent stroke in atrial fibrillation (AF) patients with a history of stroke or transient ischemic attack (TIA), a multivariable logistic regression model was used to assess the risk of in-hospital outcomes. In conducting the regression analysis, we took into account factors including age, at admission, gender, race, income level, payment status, type of admission, hospital size, teaching status of the facility, geographical location and relevant medical conditions, relevant cardiac and extracardiac comorbidities and prior history of myocardial infarction or revascularization with percutaneous coronary intervention or coronary artery bypass grafting, stroke, venous thromboembolic events, and cardiac arrest. Adjusted odds ratio (OR), 95% confidence interval (CI), and *p*-values were used to present logistic regression results. IBM SPSS Statistics 25.0 (IBM Corp., Armonk, New York, NY, USA) software was used for all statistical analyses using complex sample modules. A two-tailed *p*-value of less than 0.05 was used to determine statistical significance.

## 3. Results

### 3.1. Study Population

A retrospective analysis was conducted on a cohort of non-diabetic atrial fibrillation (AF) patients hospitalized for recurrent stroke.

### 3.2. Demographics

Out of 18,905 non-diabetic AF patients, 480 (2.5%) had prediabetes with a median age of 78 years (range 69–84), while 18,425 were non-prediabetic with a median age of 82 years (range 73–88). The prediabetic cohort was more likely to consist of male (50% vs. 45.9%) and Black patients (15.6% vs. 9.4%) compared to the non-prediabetic cohort, Table 1.

### 3.3. Risk of Recurrent Stroke

Prediabetes in AF patients hospitalized with prior stroke/TIA independently predicted a two-fold higher risk of recurrent stroke on both unadjusted (OR 2.14, 95% CI 1.72–2.66) and adjusted (adjusted for socio-demographics/comorbidities, OR 2.09, 95% CI 1.65–2.64) regression analyses (*p* < 0.001), Table 2.

*Multivariable odds of all-cause mortality were calculated adjusting for age, sex, race, income quartile, payer status, hospital bed size, location/teaching status, region, type of admission, complicated hypertension, hyperlipidemia, smoking, peripheral vascular disease, obesity, severe renal failure, prior myocardial infarction/revascularization, prior history of venous thromboembolic events, personal history of cancer, congestive heart failure, valvular heart diseases, pulmonary circulation disease, chronic obstructive pulmonary disease, chronic liver disease, AIDS, metastatic and non-metastatic cancer, arthropathies, coagulopathies, fluid electrolyte disorders, deficiency anemias, alcohol abuse, drug abuse, psychoses, hypothyroidism, other neurological disorders, and depression

### 3.4. Comorbidities

The prediabetes group was more likely to have higher Medicaid enrollees, admissions from the Northeastern/Western regions, and higher rates of hyperlipidemia, smoking, peripheral vascular disease, obesity, and chronic obstructive pulmonary disease (COPD) (all *p* < 0.05). Table 2 illustrates the co-morbidity data. 

### 3.5. Healthcare Utilization

The prediabetes cohort often required home health care (22.9% vs. 17.3%) and had higher hospital costs.

### 3.6. All-Cause Mortality

However, adjusted odds of all-cause mortality were not statistically significant in prediabetics vs. non-prediabetics (OR 0.55, 95% CI 0.19–1.56, *p* = 0.260), Table 2.

## 4. Discussion

The findings of this study on the association between prediabetes and recurrent stroke in non-diabetic AF patients have significant relevance to clinical practice. The study demonstrated that prediabetes is an independent risk factor for recurrent stroke in this population, with a two-fold higher risk compared to non-prediabetic AF patients. This highlights the importance of screening for prediabetes in AF patients with a history of stroke or TIA to identify those at higher risk for recurrent stroke. We found that the prediabetic cohort had a higher prevalence of several comorbidities, including hyperlipidemia, smoking, peripheral vascular disease, obesity, and COPD. This suggests that prediabetes is associated with a more extensive burden of cardiovascular risk factors, underscoring the need for comprehensive risk factor management in this population. Moreover, the prediabetic cohort was more likely to require home health care and had higher hospital costs, which could have implications for resource utilization in clinical practice.

Prior studies have linked prediabetes with myocardial infarction [2], stroke [8], electrocardiogram changes [9], heart failure [10], coronary heart disease, cancer, and dementia [5]. Recently, it has even been implicated as a risk factor for new-onset atrial fibrillation [11]. Even looking at the prognosis, nondiabetic patients with hyperglycemia have a worse stroke prognosis [12]. In terms of organ damage, two out of three patients with coronary artery disease exhibit compromised glucose metabolism [13]. Our study’s findings are in support of the findings by Kezerle et al. [14], that concluded that prediabetes increases stroke risk. Although, some studies also suggested both high and low glucose to cause an increase in stroke risk [15]. 

Healthcare providers may need to allocate more resources to manage the complex needs of prediabetic AF patients to optimize outcomes and contain costs. The CHA2DS2VASc score does not account for conditions that are included in metabolic syndromes like prediabetes [16]. Hence, our study’s findings can be used to facilitate decision-making in such cases. 

Our study’s findings have several implications for clinical decision-making, including the need for intensified risk factor management and lifestyle modifications, such as dietary changes and increased physical activity, to reduce the risk of recurrent stroke in prediabetic AF patients. The identification of prediabetes could prompt healthcare providers to initiate pharmacological interventions, such as metformin, to improve glucose control and presumptively reduce the risk of atherosclerosis. Deng et al. reported that metformin can improve vascular intimal thickening, calcification, and inflammation by affecting vascular smooth muscle cells, all of which are important pathological features of atherosclerosis that ultimately cause major pathological processes of stroke, angina pectoris, and myocardial infarction [17]. Furthermore, the study’s findings could inform the development of clinical practice guidelines for the management of prediabetic AF patients, potentially improving outcomes for this vulnerable population [18].

In summary, optimizing outcomes for prediabetic atrial fibrillation (AF) patients requires a multifaceted approach, including specialized care teams, patient education, structured lifestyle programs with behavioral support, meticulous medication management, rigorous blood sugar monitoring, and ECG assessments [19,20]. Addressing shared risk factors such as hypertension, dyslipidemia, and obesity is crucial to reduce stroke risk [20]. Recent literature also indicates that continuous rhythm monitoring with implantable loop recorders (ILRs) can provide valuable information for rhythm control and oral anticoagulation (OAC) decisions in AF patients who have undergone ablation. These approaches could enhance care and offer the potential for improved outcomes in these high-risk patients [21,22].

## 5. Limitations

The present study analyzed a large national database, the National Inpatient Sample (NIS), to investigate the association between prediabetes and recurrent stroke risk in patients with atrial fibrillation (AF). While the study provides important insights into the impact of prediabetes on stroke recurrence, several limitations should be considered when interpreting the findings. Firstly, the study is limited by the retrospective design and the reliance on administrative data. Although the NIS database is the largest publicly available inpatient database in the United States, it has inherent limitations due to the nature of the data collection process. The accuracy of coding and completeness of data is dependent on the quality of the documentation in the medical record, which can vary widely across hospitals and healthcare providers. Furthermore, there may be a misclassification of variables or selection bias, which could affect the results of the analysis. Secondly, the study is limited by the lack of information on the duration and severity of prediabetes. The definition of prediabetes was based on diagnostic codes in the medical record and did not include information on laboratory results, such as fasting plasma glucose or hemoglobin A1c levels. As a result, it is unclear whether patients with prediabetes had long-standing or poorly controlled hyperglycemia, which may have a greater impact on the risk of recurrent stroke. Similarly, the study did not differentiate between different types of prediabetes, such as impaired fasting glucose or impaired glucose tolerance, which may have different clinical implications. Thirdly, the duration of follow-up for the study participants was not available, given that retrospective studies focused on hospitalizations typically lack information on the follow-up period. Lastly, the study is limited by the lack of information on potential confounding factors, such as lifestyle behaviors and medication use. The NIS database does not include information on smoking status, physical activity, diet, or medication adherence, which are known risk factors for stroke and may differ between prediabetic and non-prediabetic patients. The study also did not include information on medication use, such as antithrombotic use.

## 6. Future Directives

Given the study’s revelation of prediabetes as an independent risk factor for recurrent stroke in non-diabetic AF patients, future research should prioritize prospective studies with a focus on assessing the duration and severity of prediabetes. Incorporating laboratory results, such as fasting plasma glucose and hemoglobin A1c levels, would offer a more nuanced understanding of the impact of prediabetes on stroke recurrence. To address limitations related to the retrospective design and reliance on administrative data, future investigations should consider comprehensive data collection methodologies, potentially incorporating lifestyle behaviors, medication use, and more detailed patient histories. Furthermore, efforts should be directed toward developing standardized guidelines for the management of prediabetic AF patients, integrating lifestyle modifications, intensified risk factor management, and potential pharmacological interventions. These directives aim to enhance the precision of risk stratification, improve patient outcomes, and the development of evidence-based clinical practices.

## 7. Conclusions

In conclusion, our research establishes a noteworthy and independent association between prediabetes and recurrent stroke in non-diabetic AF patients, demonstrating a twofold elevated risk in those with prediabetes. To optimize outcomes for individuals with prediabetic AF, a comprehensive approach is essential, involving specialized care teams, patient education, structured lifestyle programs with behavioral support, precise medication management, thorough blood sugar monitoring, and ECG assessments. The significance of addressing shared risk factors such as hypertension, dyslipidemia, and obesity is emphasized for the reduction of stroke risk. Recent literature suggests that continuous rhythm monitoring using implantable loop recorders (ILRs) can offer valuable information for rhythm control and oral anticoagulation (OAC) decisions in AF patients post-ablation, presenting opportunities for improved outcomes in this high-risk population.

## Figures and Tables

**Table 1:** Baseline Characteristics of Study Population.

		Prediabetes		Total Recurrent Stroke with AF (Excluding DM)	*p*-Value
		No	Yes		
		*n* = 18,425	*n* = 480	*n* = 18,905	
Age (years) at admission	Median [IQR]	82 (73–88)	78 (69–84)	82 (73–88)	<0.001
Sex	Male	45.9%	50.0%	46.0%	0.078
	Female	54.1%	50.0%	54.0%	
Race	White	80.7%	67.7%	80.3%	<0.001
	Black	9.4%	15.6%	9.6%	
	Hispanic	5.6%	11.5%	5.7%	
	Asian or Pacific Islander	2.2%	4.2%	2.2%	
Median household income national quartile for patient ZIP Code	0–25th	24.4%	25.5%	24.4%	0.543
	26–50th	25.7%	26.6%	25.8%	
	51–75th	25.7%	26.6%	25.7%	
	76–100th	24.2%	21.3%	24.1%	
Primary expected payer	Medicare	85.2%	78.1%	85.0%	<0.001
	Medicaid	2.5%	5.2%	2.5%	
	Private including HMO	9.7%	15.6%	9.8%	
Location/teaching status of hospital	Rural	6.9%		6.8%	<0.001
	Urban non-teaching	19.4%	15.6%	19.3%	
	Urban teaching	73.7%	82.3%	73.9%	
Region of hospital	Northeast	16.7%	19.8%	16.8%	<0.001
	Midwest	23.4%	17.7%	23.3%	
	South	39.5%	25.0%	39.2%	
	West	20.3%	37.5%	20.7%	
COMORBIDITIES					
Hypertension		85.2%	85.4%	85.2%	0.913
Hyperlipidemia		59.3%	74.0%	59.6%	<0.001
Smoking		37.4%	43.7%	37.6%	0.005
Peripheral vascular disease		13.0%	17.7%	13.1%	0.003
Obesity		8.0%	18.7%	8.3%	<0.001
Renal failure		19.1%	20.8%	19.1%	0.334
Prior MI		10.6%	8.3%	10.6%	0.109
Prior PCI		0.9%	4.2%	1.0%	<0.001
Prior CABG		8.9%	5.2%	8.8%	0.005
Prior VTE		7.0%	3.1%	6.9%	0.001
Cancer		17.7%	17.7%	17.7%	0.981
Congestive heart failure		26.4%	24.0%	26.3%	0.24
Valvular heart disease		18.8%	18.7%	18.8%	0.963
Chronic pulmonary disease		18.2%	21.9%	18.3%	0.042
Rheumatoid arthritis/collagen vas		3.4%	3.1%	3.4%	0.726
Coagulopathy		6.3%	8.3%	6.3%	0.071
Fluid and electrolyte disorders		26.9%	22.9%	26.8%	0.052
Deficiency Anaemias		14.1%	11.5%	14.0%	0.102
Other neurological disorders		6.0%	6.3%	6.0%	0.838
Hypothyroidism		20.2%	14.6%	20.0%	0.002
Depression		10.8%	11.5%	10.8%	0.633

*p* < 0.05 indicate statistical significance. IQR = interquartile range, HMO = health maintenance organization, MI = myocardial infarction, PCI = percutaneous coronary intervention, CABG = coronary artery bypass grafting, VTE = venous thromboembolic events.

**Table 2:** Odds of Recurrent Stroke with Prediabetes and Subsequent In-hospital Outcomes.

Outcomes		aOR	95% LL	95% UL	*p*
Odds of Recurrent Stroke	Unadjusted odds	2.14	1.72	2.66	<0.001
	Odds when adjusted for baseline demographics and hospital level characteristics	2.13	1.69	2.69	<0.001
	Odds when adjusted for baseline demographics and hospital level characteristics plus pre-existing comorbidities	2.09	1.65	2.64	<0.001
Odds of Subsequent In-hospital Mortality		0.55	0.19	1.56	0.260
		Prediabetes			
		No	Yes	Total Recurrent Stroke with AF (Excluding DM)	*p*-Value
All-cause Mortality		7.6%	4.2%	7.5%	0.005
Disposition of patient	Routine	25.0%	44.8%	25.5%	<0.001
	Transfers to short term hospitals	2.5%		2.5%	
	Other transfers incl. SNF, ICF	47.0%	26.0%	46.4%	
	Home health care	17.3%	22.9%	17.4%	
Length of stay (days)	Median [IQR]	(2–6)	(2–5)	(2–6)	0.002
Cost	Median [IQR]	(6657–17,693)	(7590–20,563)	(6661–17,776)	0.003

SNF = skilled nursing facility, ICF = intermediate care facility, AF = atrial fibrillation, DM = diabetes mellitus, aOR = adjusted odds ratio, CI = confidence interval. *p* < 0.05 indicates statistical significance.

## Data Availability

Data is available from HCUP website.
